# The effect of the universal two-child policy on medical insurance funds with a rapidly ageing population: evidence from China’s urban and rural residents’ medical insurance

**DOI:** 10.1186/s12889-021-11367-7

**Published:** 2021-07-22

**Authors:** Xinjie Zhang, Jingru Huang, Ying Luo

**Affiliations:** Department of Public Administration, School of Management, Jiangsu University, Zhenjiang, China

**Keywords:** Rapidly ageing, Universal two-child policy, Urban and rural residents’ basic medical insurance (URRBMI), Fund sustainability

## Abstract

**Background:**

With the rapid growth of the ageing population, the operating burden of China’s basic medical insurance fund is becoming increasingly heavy. To counter rapid population ageing and ameliorate a series of problems, China has adjusted its fertility policies several times. On January 1, 2016, the universal two-child policy was implemented. This study analysed the impacts of the adjustment to the fertility policy and potential improvements in fertility intention on the insured population and medical insurance fund sustainability.

**Methods:**

We used an actuarial science method and took the urban and rural residents’ basic medical insurance (URRBMI) of China, which covers most urban and rural residents, as an example to build a dynamic forecast model of population growth and a dynamic actuarial model of medical insurance funds.

**Results:**

Compared with the original policy, under the current fertility intention (40%) with the universal two-child policy, the ageing of the population structure of URRBMI participants will decline significantly after 2026, and individuals aged 65 and over will account for only 19.01% of the total participants in 2050. The occurrence of the current deficit and accumulated deficit of the URRBMI fund will be postponed for one year to 2022 and 2028, respectively. If fertility intentions continue to rise, the ageing degree of the population structure will decrease, and the deficit would be further delayed.

**Conclusions:**

The universal two-child policy is conducive to improving the degree of overall population ageing, delaying the occurrence of a URRBMI fund deficit, and improving the sustainability of URRBMI funds. If fertility intention increases, the effects would be stronger. However, since the adjustment of the universal two-child policy has a certain time lag, it will take time to demonstrate this impact. Therefore, while actively promoting the universal two-child policy, other measures should be taken, such as improving the fertility desire among couples of childbearing age and reforming medical insurance payment methods.

## Background

Ageing populations are a common problem for countries around the world [1]. It is an inevitable population trend at a certain stage of social and economic development [2]. In 1999, the population over 60 years old accounted for 10% of the total population in China, but it only took eight years for this fraction to increase to 17.3%. The speed and scale of ageing in China’s society are unprecedented worldwide. Judging from the development trajectory of most countries with ageing populations, continued population ageing will profoundly change the economy, society, and medical insurance system [3–5]. Along with the rapid ageing of the population, the medical needs of the elderly population are increasing [6]. According to the Statistical Yearbook of China’s Health and Family Planning 2019, in 2013, the gap between the two-week consultation rate and hospitalization rate of elderly people aged 65 and above in the surveyed areas and those aged 15–34 was as high as 6–8 times, and this gap is still on the rise. In China, influenced by the continuous growth of medical needs of the elderly as the population ages and by the ongoing improvements of medical security level, the utilization rate of medical insurance funds and the risk of fund expenditure and depletion both continue to increase. Moreover, the ageing population can indirectly reduce inputs to medical insurance funds through changes in the population structure [7]. With a rapidly ageing population, the growth rate of medical insurance fund expenditure is greater than that of medical insurance fund income [8]. If this situation continues, the sustainability of medical insurance funds will ultimately be affected in the future [9].

In China, the basic medical insurance system consists of two parts: urban employees’ basic medical insurance (UEBMI) and urban and rural residents’ basic medical insurance (URRBMI). URRBMI is the result of a merger of urban residents’ basic medical insurance (URBMI) and the new rural cooperative medical care system (NCMS) in 2016 and mainly provides coverage for urban and rural residents who do not take part in UEBMI (generally minors and jobless residents). In 2020, a total of 1016.76 million people were insured by URRBMI, accounting for 74.69% of the total population insured by China’s basic medical insurance, which is a significant proportion. However, as URRBMI fundraising comes from financial subsidies and personal contributions, financial subsidies are the main source of fundraising, while personal contributions are auxiliary. Additionally, as the compensation level has continuously increased in recent years, URRBMI fund expenditures continue to increase. While UEBMI funds operate well nationwide, at present, some provincial (regional and municipal) URRBMI funds have appeared to reach or are facing the risk of deficit [10]. Rapid ageing undoubtedly worsens the situation, increasing the deficit risk of URRBMI funds and affecting basic medical insurance fund sustainability.

An aging population, decreases in the fertility rate, and other factors can promote the adjustment of the national fertility policy [11, 12]. In response to the rapid aging of its population, in 2013, China initiated the implementation of a selective two-child policy to replace the original strict one-child policy, allowing couples to have two children if one parent was an only child. However, because the effect of the policy was not obvious, a universal two-child policy was fully implemented on January 1, 2016, which allowed each couple to have two children. Then on May 31, 2021, the Chinese government further proposed to implement the policy that one couple can have three children and supporting measures. The successive adjustment of the fertility policy fully demonstrates the stark reality of China’s rapidly aging population. Because such a fertility policy can affect the total population and population structure [13], the government expects to reduce the aging population problem through the universal two-child policy and achieve many other positive impacts, such as weakening the deficit risk of medical insurance funds.

To investigate how to increase the sustainability of funds or weaken the deficit risk of funds, some studies have carried out quantitative research based on the perspective of balancing fund expenditure and income [8, 14–16]. Demographic factors affecting the balance of medical insurance funds, such as the ageing population, have been the focus of several scholars [7, 8]. However, to some extent, the adjustment of the fertility policy can relieve the pressure generated by a labour shortage and an ageing population [17, 18]. To date, most studies have focused on the relationship between fertility policies and social pensions [19–21], and few scholars have focused on the connection between the adjustment of fertility policies and medical insurance. In China, under the background of the continuous adjustment of the fertility policy, a few scholars have begun to focus their research on the relationship between the adjustment of the fertility policy and the sustainability of medical insurance funds, successively researching the impact of the selective two-child policy and the universal two-child policy on medical insurance funds. In addition, compared with URRBMI, the sustainability concerns regarding the UEBMI fund have attracted more attention from scholars, and the forecast horizon of much of this research is 2050. These studies concluded that the adjustment of the fertility policy could delay the occurrence of the deficit of the UEBMI fund and reduce the deficit amount to a certain extent; this has been generally recognized by scholars [20–24].

At present, most studies are based on a single perspective—the issues of an ageing population or fertility policy adjustments—to research the sustainability of medical insurance funds. However, few scholars have combined the two. Moreover, in China, few scholars have paid attention to the sustainability of the URRBMI fund. Faced with the rapidly ageing population, will China’s universal two-child fertility policy that has been implemented for more than five years ease the ageing population process, promote the balance of URRBMI fund income and expenditure, and realize the sustainable operation of the fund? To provide theoretical answers to these questions, this study took China’s URRBMI as an example to study the impact of fertility policy adjustment and changes in fertility intentions on the sustainability of medical insurance funds and the ageing population structure from the realistic background of the rapidly ageing population by constructing a dynamic population growth forecast model and an actuarial model of the URRBMI fund. Ultimately, this study provides corresponding countermeasures for improving the sustainability of the URRBMI fund.

## Methods

### Model design

Actuarial science studies the effects of uncertain future events. As an important application of actuarial science, medical insurance mainly includes the experiential data of the population, society, economy, and fund operation to forecast and evaluate fund revenues and expenditures in the future to analyse the solvency, risk status, and long-, medium- and short-term financial status of medical insurance funds.

A dynamic actuarial model of the URRBMI fund was established to evaluate the sustainability of URRBMI fund operation in the target period. The model includes two parts: the income and expenditure forecast model and the accumulated balance model. The former is mainly used to analyse the URRBMI fund income and expenditure, and it can be divided into the income forecast model and expenditure forecast model. The latter is used to investigate whether the URRBMI fund has the ability to operate sustainably. When the accumulated balance is greater than 0, the fund can still self-regulate, and its overall operation is stable. However, if the accumulated balance is less than 0, the URRBMI fund is no longer capable of providing adequate medical security and running sustainably.

#### Income forecast model of the funds

Since 2013, provinces have started to integrate the URBMI and NCMS according to the national requirements to establish a unified URRBMI, but this integration is not yet fully complete. On this basis, this study adopted a separate calculation method to obtain the URRBMI fund income. This means that the income of the URRBMI fund in year t in this study should be equal to the sum of the income in year t of the NCMS fund and that of the URBMI fund. The income of the NCMS fund in year t is equal to the number of NCMS participants in year t multiplied by the per capita financing standard in year t. The income of the URBMI fund in year t is equal to the number of URBMI participants in year t multiplied by the per capita financing standard in year t, as shown in formula (1):

1 $$ {\displaystyle \begin{array}{l}{(AI)}_t=\left(\sum \limits_{x=0}^{100}{N}_{t,x}^{r,m}+\sum \limits_{x=0}^{100}{N}_{t,x}^{r,f}\right)\times {(PI)}_t^r+\left(\sum \limits_{x=0}^{100}{N}_{t,x}^{u,m}+\sum \limits_{x=0}^{100}{N}_{t,x}^{u,f}\right)\times {(PI)}_t^u\\ {}=\left(\sum \limits_{x=0}^{100}{N}_{t,x}^{r,m}+\sum \limits_{x=0}^{100}{N}_{t,x}^{r,f}\right)\times {\left[{(PA)}_{2020}^r\times \prod \limits_{i=2021}^t\left(1+{\alpha}_i^r\right)+{(PB)}_{2020}^r\times \prod \limits_{i=2021}^t\left(1+{\beta}_i^r\right)\right]}_t^r\\ {}+\left(\sum \limits_{x=0}^{100}{N}_{t,x}^{u,m}+\sum \limits_{x=0}^{100}{N}_{t,x}^{u,f}\right)\times {\left[{(PA)}_{2020}^u\times \prod \limits_{i=2021}^t\left(1+{\alpha}_i^u\right)+{(PB)}_{2020}^u\times \prod \limits_{i=2021}^t\left(1+{\beta}_i^u\right)\right]}_t^u\end{array}} $$

Where (*AI*)_*t*_ is the URRBMI fund income in year t. ${N}_{t,x}^{r,m}$ and ${N}_{t,x}^{r,f}$ are the populations of males and females of X years of age who were insured by the NCMS in the year t, respectively. ${N}_{t,x}^{u,m}$ and ${N}_{t,x}^{u,f}$ are the populations of males and females of X years of age, respectively, who were insured by the URBMI in year t. (*PI*)_*t*_^*r*^ and (*PI*)_*t*_^*u*^ are the per capita financing standards in year t of the NCMS and URBMI, respectively. ${(PA)}_{2020}^r$ and ${(PA)}_{2020}^u$ are the subsidies of per capita financing of the NCMS and URBMI, respectively, from public finance in 2020. ${(PB)}_{2020}^r$ and ${(PB)}_{2020}^u$ are the individual contribution amounts of the insured population of the NCMS and URBMI, respectively, in 2020. *α*_*i*_^*r*^ and *α*_*i*_^*u*^ are the average annual growth rates of the financial subsidy part in the per capita financing standard of the NCMS and URBMI, respectively, in year i (*i* ≤ *t*). ${\beta}_i^r$ and ${\beta}_i^u$ are the average annual growth rates of the individual contribution part in the per capita financing standard of the NCMS and URBMI, respectively.

#### Expenditure forecast model of the funds

The URRBMI fund expenditure in year t should be equal to the sum of the expenditure in year t of the NCMS fund and URBMI fund; this is the same principle used to construct the income forecast model of the fund. The NCMS fund expenditure in year t is equal to the insured population of the NCMS in year t multiplied by the corresponding per capita compensation expenditure, and the URBMI fund expenditure in year t is equal to the insured population of the URBMI in year t multiplied by the corresponding per capita compensation expenditure, as shown in formula (2):

2 $$ {\displaystyle \begin{array}{l}{(AC)}_t=\left(\sum \limits_{x=0}^{100}{N}_{t,x}^{r,m}+\sum \limits_{x=0}^{100}{N}_{t,x}^{r,f}\right)\times {\left(\overline{PC}\right)}_t^r+\left(\sum \limits_{x=0}^{100}{N}_{t,x}^{u,m}+\sum \limits_{x=0}^{100}{N}_{t,x}^{u,f}\right)\times {\left(\overline{PC}\right)}_t^u\\ {}\hfill =\left(\sum \limits_{x=0}^{100}{N}_{t,x}^{r,m}+\sum \limits_{x=0}^{100}{N}_{t,x}^{r,f}\right)\times {\left(\overline{MC}\right)}_{2020}^r\times \prod \limits_{i=2021}^t\left(1+{k}_i^r\right)\times {U}_{2020}^r\hfill \\ {}\hfill +\left(\sum \limits_{x=0}^{100}{N}_{t,x}^{u,m}+\sum \limits_{x=0}^{100}{N}_{t,x}^{u,f}\right)\times {\left(\overline{MC}\right)}_{2020}^u\times \prod \limits_{i=2021}^t\left(1+{k}_i^u\right)\times {U}_{2020}^u\hfill \end{array}} $$

(*AC*)_*t*_ represents the URRBMI fund expenditure in year t. ${\left(\overline{PC}\right)}_t^r$ and ${\left(\overline{PC}\right)}_t^u$ are the per capita compensation expenditures in year t of the NCMS and URBMI, respectively. ${\left(\overline{PC}\right)}_t^r$ is equal to the per capita medical expenses in year t of the NCMS multiplied by the actual compensation ratio of the NCMS (${\left(\overline{PC}\right)}_t^r={\left(\overline{MC}\right)}_t^r\times {U}_t^r$). ${\left(\overline{PC}\right)}_t^u$ is equal to the per capita medical expenses in year t of the URBMI multiplied by the actual compensation ratio of the URBMI (${\left(\overline{PC}\right)}_t^u={\left(\overline{MC}\right)}_t^u\times {U}_t^u$).

The reason for choosing the actual compensation ratio is that according to previous studies [25, 26], the actual compensation ratio can reflect the actual guarantee level of the URRBMI more accurately than the policy compensation ratio. This is also applicable for the horizontal comparison of guarantee level between different systems, different regions and groups of people. ${k}_i^r$ and ${k}_i^u$ are the average annual growth rates of the per capita medical expenses of the NCMS and URBMI, respectively, in year i. The meanings of the other parameters are the same as above.

#### Accumulated balance (or accumulated deficit) forecast model

The accumulated balance (or accumulated deficit) of the URRBMI fund in year t is equal to the sum of the accumulated balance (or accumulated deficit) in year t of the NCMS fund and URBMI fund. The former is equal to the sum in the NCMS fund of the accumulated balance (or accumulated deficit) of year t-1 and the current balance (or current deficit) in year t, while the latter is equal to the sum in the URBMI fund of the accumulated balance (or accumulated deficit) in year t-1 and the current balance (or current deficit) in year t, as shown in formula (3):

3 $$ {\displaystyle \begin{array}{l}{S}_t={S}_t^r+{S}_t^u\\ {}=\left({S}_{t-1}^r+{S}_{t-1}^u\right)\times \left(1+{\sigma}_1\right)+\left\{\left[{(AI)}_t^r-{(AC)}_t^r\right]+\left[{(AI)}_t^u-{(AC)}_t^u\right]\right\}\times \left(1+{\sigma}_2\right)\end{array}} $$

${S}_t^r$and ${S}_t^u$ are the accumulated balances (or accumulated deficits) of the NCMS fund and URBMI fund, respectively, in year t. ${(AI)}_t^r-{(AC)}_t^r$ and ${(AI)}_t^u-{(AC)}_t^u$ are the current balances (or current deficits) of the NCMS fund and URBMI fund, respectively, in year t. *σ*_1_ and *σ*_2_ are the interest-bearing interest rates of the accumulated balance and current balance during the forecast period, respectively. According to these hypotheses, *σ* = *σ*_1_ = *σ*_2_. Because the accumulated balance (or accumulated deficit) and the current balance (or current deficit) may not coincide with each other, the accumulated balance (or accumulated deficit) forecast model of the URRBMI fund should include the following three situations:

##### Situation 1

When the current balance is greater than 0 and the accumulated balance is also greater than 0. Assume that when t = 2021,2022,...,t + x-1, the current balance and accumulated balance of the URRBMI fund are both greater than zero. Therefore, the accumulated balance of the URRBMI fund in year t should be equal to the sum of the current balance of the URRBMI fund (including the amount of interest) in year t and the accumulated balance of the URRBMI fund (including the amount of interest) of year t-1, as shown in formula (4):

4 $$ {S}_t=\sum \limits_{m=2021}^t\left\{\left[{(AI)}_m^r-{(AC)}_m^r\right]\times {\left(1+\sigma \right)}^{t-m+1}\right\}+\sum \limits_{m\hbox{'}=2021}^t\left\{\left[{(AI)}_{m\hbox{'}}^u-{(AC)}_{m\hbox{'}}^u\right]\times {\left(1+\sigma \right)}^{t-m\hbox{'}+1}\right\}+\left({S}_{2020}^r+{S}_{2020}^u\right)\times {\left(1+\sigma \right)}^{t-2020} $$

##### Situation 2

When the current balance is not greater than 0 and the accumulated balance is greater than 0. Assume that when t = t + x,t + x + 1,...,t + y, the current balance of the URRBMI fund begins as less than 0, which means a deficit for the current period. However, at this time, the accumulated balance is still greater than 0. Therefore, at this time, interest would only accrue on the accumulated balance, and no interest would accrue on the current deficit, as shown in formula (5):

5 $$ {\displaystyle \begin{array}{l}{S}_t=\sum \limits_{m=2021}^{t+x}\left\{\left[{(AI)}_m^r-{(AC)}_m^r\right]\times {\left(1+\sigma \right)}^{t+x-m+1}\right\}+\sum \limits_{m=t+x+1}^{t+y}\left[{(AI)}_m^r-{(AC)}_m^r\right]\\ {}+\sum \limits_{m^{\prime }=2021}^{t+x}\left\{\left[{(AI)}_{m^{\prime}}^u-{(AC)}_{m^{\prime}}^u\right]\times {\left(1+\sigma \right)}^{t+x-{m}^{\prime }+1}\right\}+\sum \limits_{m^{\prime }=t+x+1}^{t+y}\left[{(AI)}_{m^{\prime}}^u-{(AC)}_{m^{\prime}}^u\right]+\left({S}_{2020}^r+{S}_{2020}^u\right)\times {\left(1+\sigma \right)}^{t+x-2020}\end{array}} $$

##### Situation 3

When the current balance is not greater than 0 and the accumulated balance is also not greater than 0. Suppose that when t = t + y + 1,t + y + 2,...,2030/2050, the current balance and accumulated balance of the URRBMI fund all begin as less than 0, which means that there are current deficit and accumulated deficit successively. Then, neither accrues interest at this time, as shown in formula (6):

6 $$ {\displaystyle \begin{array}{l}{S}_t=\sum \limits_{m=2021}^{t+y+1}\left\{\left[{(AI)}_m^r-{(AC)}_m^r\right]\times {\left(1+\sigma \right)}^{t+y+1-m}\right\}+\sum \limits_{m=t+y+2}^{2030}\left[{(AI)}_m^r-{(AC)}_m^r\right]\\ {}+\sum \limits_{m^{\prime }=2021}^{t+y+1}\left\{\left[{(AI)}_{m^{\prime}}^u-{(AC)}_{m^{\prime}}^u\right]\times {\left(1+\sigma \right)}^{t+y+1-{m}^{\prime }}\right\}+\sum \limits_{m^{\prime }=t+y+2}^{2030}\left[{(AI)}_{m^{\prime}}^u-{(AC)}_{m^{\prime}}^u\right]+\left({S}_{2020}^r+{S}_{2020}^u\right)\times {\left(1+\sigma \right)}^{t+y-2019}\end{array}} $$

### Data processing and parameter specification

The data in this study were mainly sourced from national data of China such as the Sixth National Census and annual National Statistical Yearbook. This study also in the light of research [27–31] in China to modify the relevant data and determine the following parameters.

#### Sizes of the population insured by the NCMS and URBMI

According to the model, the sizes of the population insured by the NCMS and URBMI in the forecast period can be calculated. Since both the NCMS and the URBMI have achieved comprehensive coverage, this study assumed that the participation rate of both is 100%. The specific calculation process is as follows. 1) Multiply the population data in the Sixth National Census divided according to age (0–100 years old), sex, and urban and rural residence by the survival probability of the corresponding year to obtain the number of natural population growth for the next year. Referring to the requirements for the age of the population in the Sixth National Census data, this study set the value range of the age of the paying population as 0–100 years old, while individuals 100 years and older are counted as being 100 years old. Besides, this study used the JPOP-1 method for smoothing and employed a survival probability = 1-crude death rate. 2) Multiply the numbers of women of childbearing age obtained in the previous step (divided into urban and rural areas) by the birth probability and sum the results to obtain the population of 0-year-old infants in both urban and rural areas. Then, combine the newborn sex ratio at birth to calculate the newborn population by sex. 3) Calculate the registered population by age, gender, and urban and rural residence in accordance with the household registration urbanization rate. Although urbanization has encouraged some rural residents to work and live in cities and participate in the URBMI, some urban residents have also been included in the management of the NCMS for reasons such as marriage or the coordination of medical insurance between urban and rural areas. Overall, the population insured by the NCMS is the same as the registered agricultural population. Combined with scholars’ research [27] and the actual urbanization of the registered population in China, this study assumed that the urbanization rate of the registered population will increase at the current level at an average annual rate of 1% and reach a peak of 75% in 2050. 4) Subtract the number of UEBMI participants from the number of urban registered populations to obtain the number of URBMI participants. The number of UEBMI participants is equal to the sum of the employed population and the retired population of the UEBMI. The number of employed people is equal to the product of the number of urban registered population, corresponding year employment rate, and UEBMI participation rate. The retired population can be calculated by the cohort element method. In general, this study obtained the number of participants of the NCMS and the URBMI by age.

#### Total fertility rate

Affected by the economic development level, fertility desire, and other factors, the total fertility rate of urban and rural residents in China has continued to decline in recent years. According to the Sixth National Census data, China’s total fertility rate in 2010 was 1.18, including 0.98 in urban areas and 1.44 in rural areas, both significantly lower than the internationally recognized population replacement level of 2.1. In 2013, the Third Plenary Session of the 18th CPC Central Committee put forward a policy allowing couples to have two children if one parent was an only child, called the selective two-child policy. However, by the end of May 2015, only 1.45 million couples had applied to have another child, accounting for 13% of the amounts of couples applicable to the adjusted policy. Given this, the Fifth Plenary Session of the 18th CPC Central Committee in 2015 proposed the universal two-child policy, also called “the full implementation of a couple can have two children” policy, and put it into effect on January 1, 2016. Considering that scholars generally believed that the total fertility rate calculated based on the data in Sixth National Census is low, this study used the method of previous scholars [28] to revise the total fertility rate. The revised total fertility rate in China was 1.27, including 1.12 in urban areas and 1.42 in rural areas. Finally, according to the “421” family microsimulation model [29], this study calculated the total fertility rates of urban and rural residents under different fertility policies and different levels of fertility desire.

#### Per capita financing standard

According to the opinions on the integration of the URRBMI issued by the State Council in 2016, the URBMI and NCMS adopt the same financing standards after Integration. However, considering the stability of the system’s development of cohesion, financing growth, and other factors, this study chose the NCMS, which has been established for a long time and has had a relatively stable operation, as a reference to set the corresponding parameters. This means that the average annual growth rate of the per capita financing standard for the URRBMI during the forecast period was set with reference to the average annual growth rate of the per capita disposable income of rural residents from 2010 to 2019. Other parameters were set according to the difference between the two. Additionally, per the National Statistical Yearbook, the average annual growth rate of farmers’ per capita disposable income was 9.68% from 2010 to 2019, slightly lower than the average annual growth rate of per capita GDP (9.74%) in the same period. Considering the current situation of normal economic development in the post-epidemic era, the study assumed that the per capita disposable income of rural residents in China will grow at an average annual rate of 10% from 2021 to 2025 and then decrease by 0.5 percentage points every five years.

#### Standard for per capita compensation expenditure

According to the model design, the per capita compensation expenditure should be equal to the per capita medical expenses multiplied by the corresponding actual compensation ratio. In this study, the “growth factor” method was used to analyse the factors affecting the growth of the medical expenses of the NCMS and URBMI. To research the impact of the universal two-child policy adjustment, that is, the change in the demographic factor, on the sustainability of medical insurance funds, this study specifically separated the demographic factor from the whole and assumed that it is independent of non-demographic factors. Specifically, demographic factor refers to the increase in medical expenses caused by the increase in population size and the change in the age structure. This study calculated the demographic factor by the insured population by age and the corresponding medical consumption weight index of each age group. Other non-demographic factors were those other than demographic factors influencing the growth of per capita medical expenses, such as the growth of per capita disposable income of urban and rural residents, the improvement of medical technology, etc. According to previous studies [30, 31], combined with the previous setting of per capita financing standards, this study assumed that the average annual growth rate without demographic factors was 1 percentage point faster than the average annual growth rate of per capita disposable income of rural residents in the same period.

#### Bank interest rate

According to the notice on strengthening the financial management of social insurance funds issued by the Ministry of Finance of China in 2003, the interest of social insurance funds should be calculated by the preferential interest rate stipulated by the People’s Bank of China, namely, the interest rate for three-month fixed deposits. Therefore, this study assumed that the current balance and the accumulated balance of the URRBMI fund are calculated by the latest three months regularly stored at a predetermined rate (1.1%), which was published by the People’s Bank of China on October 24, 2015.

The summary of the aforementioned key parameters is shown in Table 1.

**Table 1:** The summary of main parameters

Parameters	Reference values	Settings
Participation rate of URRBMI	≥95% (2020 year)	100%
Household registration urbanization rate	45.4% (2020 year)	The average annual growth rate is 1%, reaching a peak of 75% in 2050
Per capita disposable income of rural residents	16,021 yuan (2019 year)	Between 2021 and 2025, the average annual growth rate will be 10%, followed by a decrease of 0.5% every five years
Bank interest rate	1.1% (2015 year)	Remain unchanged during the forecast period
Per capita financing standard	781 yuan (2019 year)	Set regarding the growth rate of rural residents’ per capita disposable income
Per capita medical expenses	1405.7 yuan (2019 year)	Decomposition calculation using “growth factor” method
Actual compensation ratio	56.86% (2019 year)	Remain unchanged during the forecast period

**Note:** Affected by the COVID-19, the data in 2020 will fluctuate greatly, so some of the values use the data in 2019. The data are all sourced from the official websites of the National Bureau of Statistics and the National Healthcare Security Administration in China, and some are further calculated by the author based on the official website data

### Measurement process

The selective two-child policy had been implemented for less than two years before it was fully replaced by the universal two-child policy. Therefore, in the subsequent measurement, this study used the strict one-child policy, which can also be called the original policy, as the reference for the subsequent policy adjustment. Although the basic medical insurance system characterized by a pay-as-you-go scheme is a short-term forecast project, considering the time-lag effect of the adjusted fertility policy, 2030 and 2050 were chosen as the endpoints for the analysis.

It has been five years since the universal two-child policy was officially implemented in 2016. Although the number of births rose to 17.86 million in 2016, it then began to decline. The numbers of births in 2017, 2018, and 2019 were 17.23 million, 15.23 million, and 14.65 million, respectively, indicating a significant decline in the effect of the policy. Low fertility desire is undoubtedly a key factor affecting the number of births. Drawing on the study of scholars [32], this study set the current fertility intentions of females in accordance with the universal two-child policy at 40%. Moreover, childbearing willingness may fluctuate in the future, affected by factors such as financial support and maternity security. This study used the URRBMI fund operation under the strict one-child policy as a reference and measured the effect of the universal two-child policy when the fertility intention was 20, 40, 60, 80 and 100%. In addition, we analysed the influence on the URRBMI fund under different fertility policies and fertility intentions to comprehensively analyse the impact of the fertility policy adjustment.

## Results

### Relevant prediction results for URRBMI participants

**Fig. 1:**
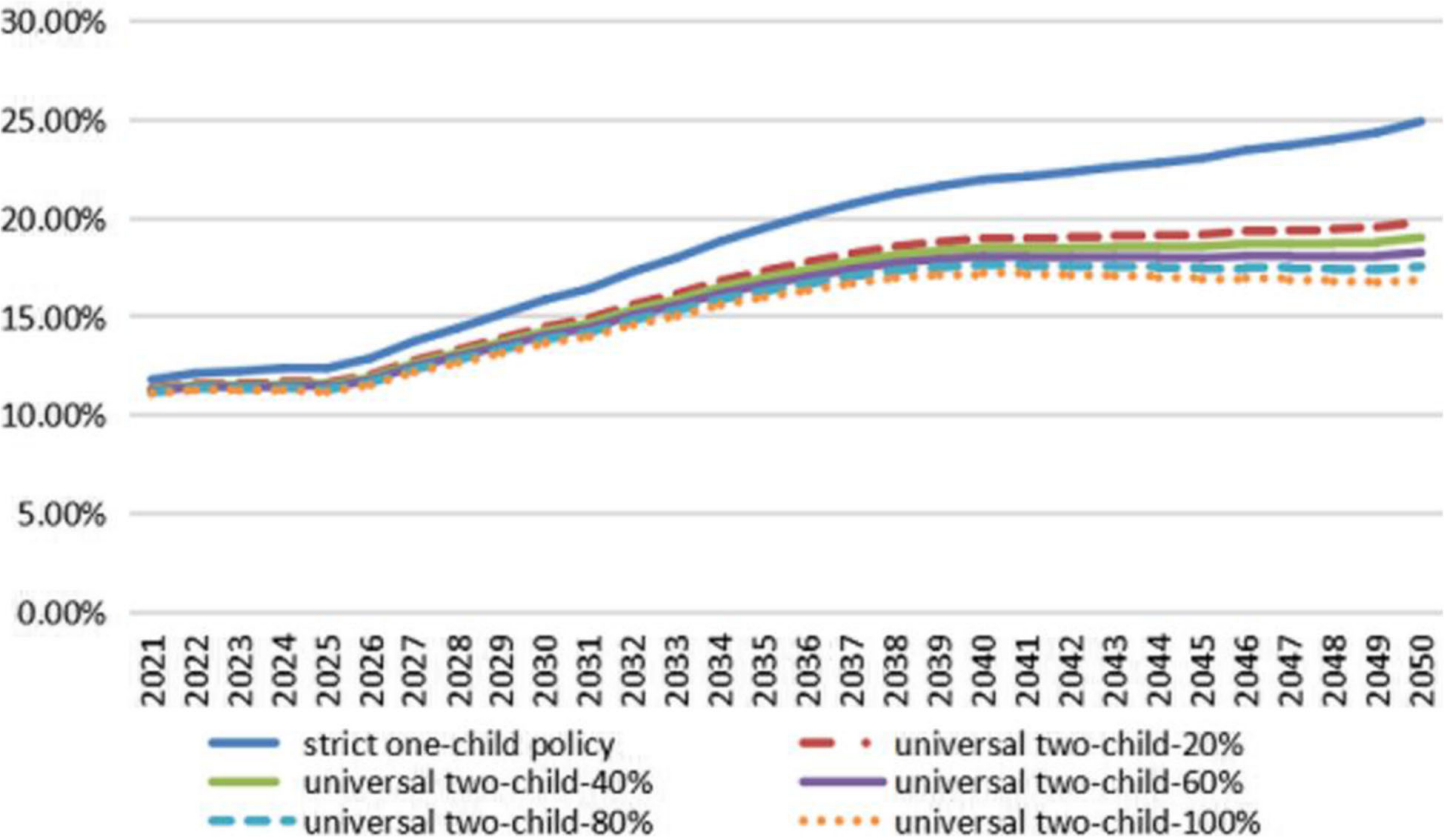
The proportion of URRBMI participants aged 65 and over in China. Note: universal two-child-20% in the figure represents the effect of the universal two-child policy under 20% fertility intention, and so on

Figure 1 reflects the changes in the proportion of the population aged 65 and over in China from 2021 to 2050 under different fertility policies and fertility intentions. As shown in Fig. 1, if the fertility policy had not been adjusted, under the strict one-child policy, the population aged 65 and over in the URRBMI would have continued to grow rapidly after 2021 and would have accounted for more than 20% in 2036, reaching a maximum of 24.92% in 2050.

However, under the current universal two-child policy and fertility intention of 40%, the ageing of the population structure will decline significantly after 2026, and individuals aged 65 and over will account for only 19.01% of the total population in 2050. It is obvious from Fig. 1 that with an increase in intention to have children, the population structure will continue to improve, and the growth rate will slow down significantly during the forecast period. When fertility willingness reaches 80%, the proportion of the population aged 65 and over will begin to decline after reaching its peak in 2046 (17.5%) during the forecast period.

**Fig. 2:**
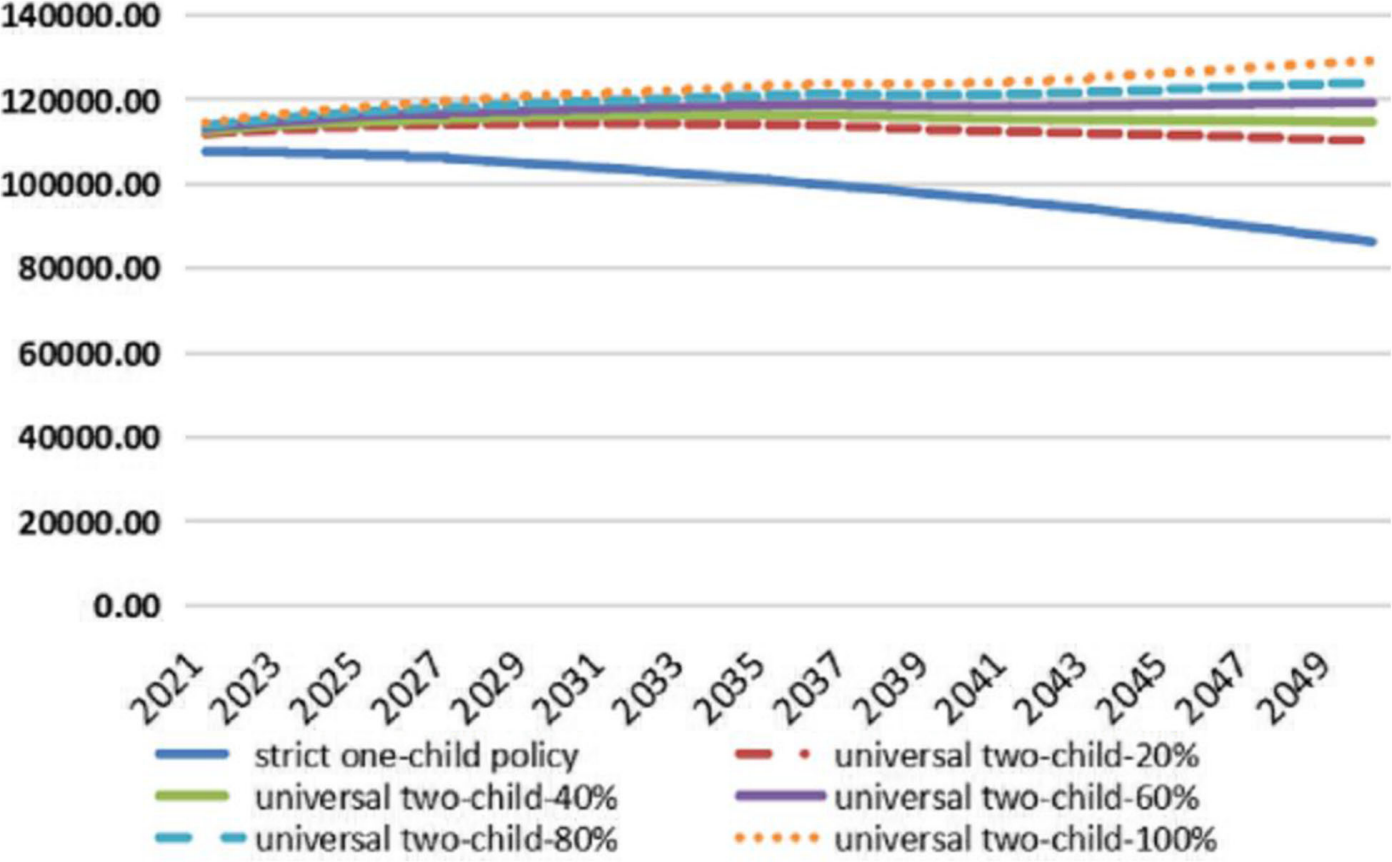
The number of URRBMI participants in China (unit: ten thousand)

Figure 2 reflects the changes in the number of URRBMI participants in China from 2021 to 2050 under different fertility policies and fertility intentions. Figure 2 shows that under the strict one-child policy, the number of URRBMI participants would show a significant downward trend after 2021. When the childbearing willingness reaches 40% under the universal two-child policy, the URRBMI participants have a slow growth rate during the forecast period. However, there is a clear upward trend and an extension of the forecast period, and the population gap between the two becomes more obvious. This is mainly because the fertility policy adjustment has a certain time lag, which is also an important reason for the extension of this prediction. It can also be seen from Fig. 2 that if fertility intention further increases to 60, 80, and 100%, the number of URRBMI participants will increase significantly, and this effect becomes more pronounced as the forecast period extends. In addition, if the willingness to give birth can be increased to 60% and over during the forecast period, the number of URRBMI participants will maintain an overall upward trend during the forecast period.

### Operation of the URRBMI fund under the strict one-child policy

Combining the aforementioned Figs. 1 and 2, it can be seen that under the strict one-child policy, the number of URRBMI participants would have shown a significant downward trend, and the population structure would have aged significantly. Around 2050, the proportion of elderly people aged 65 and over would have reached 24.92%. Under this demographic trend, as shown in Table 2, the income of China’s URRBMI fund would have increased from 1067.362 billion yuan in 2021 to 2371.367 billion yuan in 2030 and 9439.809 billion yuan in 2050. During the forecast period, the average annual growth rates of fund income and expenditure would have been 7.81 and 9.67%, respectively, resulting in the fund’s current deficit in 2021 and accumulated deficit in 2027. By 2050, the accumulated deficit would have amounted to 54,369.347 billion yuan, and the accumulated balance rate would have reached − 575.96%.

**Table 2:** Operation of China’s URRBMI fund under the strict one-child policy

Year	Fund income (Billion)	Fund expenditure (Billion)	Current balance (Billion)	Accumulated balance (Billion)	Accumulated balance rate (%)
2021	1067.362	1067.839	−0.478	527.463	49.42
2022	1168.377	1191.951	−23.573	509.692	43.62
2023	1277.936	1326.617	−48.680	466.618	36.51
2024	1397.312	1478.187	−80.875	390.876	27.97
2025	1527.287	1642.343	− 115.056	280.120	18.34
2030	2371.367	2820.293	− 448.926	−1194.312	−50.36
2035	3488.818	4573.730	− 1084.912	− 5184.605	− 148.61
2040	5077.579	7239.499	− 2161.920	−13,640.709	− 268.65
2045	6965.448	10,723.183	− 3757.736	−28,973.696	− 415.96
2050	9439.809	15,520.955	− 6081.146	−54,369.347	−575.96

**Note:** (a) Due to space limitations, this study only reports the simulation results of some years in the table, and the same applies below. (b) When the current balance or accumulated balance becomes negative, it means that the URRBMI fund has a current deficit or an accumulated deficit; that is, the fund has a payment risk (accumulated balance rate = accumulated balance/current URRBMI funds income * 100%)

**Source:** The author of this manuscript calculated the data based on the collected data and actuarial models

### Operation of the URRBMI fund under the universal two-child policy with 40% fertility intention

Combining Figs. 1 and 2, it can be seen that under the universal two-child policy with 40% fertility intention, the number of URRBMI participants in the country increases significantly compared with the original policy, and this upward trend becomes more significant with the extension of the forecast period. Moreover, the age structure of the insured population of URRBMI also improves significantly, and the proportion of the elderly population aged 65 and above drops significantly. Affected by this demographic trend, the financial status of the URRBMI fund also improves to some extent during the forecast period. As shown in Table 3, after the implementation of the universal two-child policy with 40% fertility intention, the average annual growth rate of the URRBMI fund income during the forecast period is 8.84%, and the average annual growth rate of fund expenditure is 10.19%. Compared with the strict one-child policy, the former increases by 1.03 percentage points, and the latter increases by 0.52 percentage points. The gap between the URRBMI fund income and expenditure shrinks during the forecast period, which also causes the amount and rate of increase in the current deficit and the accumulated deficit to decline. In the forecast period of 2050, the accumulated deficit will reach a maximum of 48,569.366 billion yuan, which is a decrease of 5799.981 billion yuan compared with the original policy. The accumulated balance ratio will be − 354.98%, which is a decrease of 220.98% compared with the original policy. Hence, the fund reserve capacity will be enhanced.

**Table 3:** Operation of China’s URRBMI fund under the universal two-child-40% policy

Year	Operation of the URRBMI fund					Change rate compared with original policy			
	Fund income (Billion)	Fund expenditure (Billion)	Current balance (Billion)	Accumulated balance (Billion)	Accumulated balance rate (%)	Income (%)	Expenditure (%)	Current balance (%)	Accumulated balance (%)
2021	1172.448	1161.870	10.578	576.107	49.14	9.85	8.81	− 2312.97	9.22
2022	1292.010	1297.980	−5.969	576.409	44.61	10.58	8.90	−74.68	13.09
2023	1422.761	1446.404	−23.643	559.106	39.30	11.33	9.03	−51.43	19.82
2024	1566.365	1614.042	−47.677	517.580	33.04	12.10	9.19	−41.05	32.42
2025	1724.042	1796.639	−72.598	450.676	26.14	12.88	9.39	−36.90	60.89
2030	2772.331	3117.769	− 345.438	−633.924	−22.87	16.91	10.55	−23.05	−46.92
2035	4235.749	5125.733	− 889.983	− 3844.560	−90.76	21.41	12.07	−17.97	−25.85
2040	6500.392	8388.903	− 1888.511	−11,077.908	− 170.42	28.02	15.88	−12.65	− 18.79
2045	9470.756	12,923.833	− 3453.077	−24,941.098	− 263.35	35.97	20.52	−8.11	−13.92
2050	13,682.381	19,367.284	− 5684.903	−48,569.366	−354.98	44.94	24.78	−6.52	−10.67

### Operation of the URRBMI fund under different fertility policies and fertility intentions

Combining Figs. 1 and 2, it can be seen that with an increase in the willingness of couples to have children in line with the universal two-child policy, the number of URRBMI participants continues to increase, and the proportion of elderly people aged 65 and above continues to decrease. However, because the adjustment of the fertility policy has a certain time lag, this effect only begins to appear after one fertility cycle. The opposite is true when the willingness to have children decreases. Table 4 shows that increasing the intention to give birth under the universal two-child policy delays the occurrence of an accumulated deficit of the medical insurance fund. For example, if fertility intention under the universal two-child policy increases to 40% of the current stage, the current deficit and accumulated deficit occurrence points are delayed by 1 year compared to under the strict one-child policy. At the same time, in 2030, the accumulated deficit of the URRBMI fund will fall from 1194.312 billion yuan under the strict one-child policy to 633.924 billion yuan, and in 2050, it will fall from 54,369.347 billion yuan to 48,569.366 billion yuan. If fertility intention under the universal two-child policy to give birth is further increased during the forecast period, the time for the URRBMI fund deficit is further delayed, and the accumulated deficit is further reduced. The sustainable operation of the fund will be significantly improved.

**Table 4:** Operation of the URRBMI fund under different fertility policies and fertility intentions

Situation	Time of occurrence of current deficit (Year)	Time of occurrence of accumulated deficit (Year)	Amount at the time of occurrence of deficit (Billion)		Time delay (Year)		2021–2030 Accumulated balance (Billion)	2021–2050 Accumulated balance (Billion)
			Current balance	Accumulated balance	Current balance	Accumulated balance		
Strict one-child	2021	2027	−0.478	−99.166	–	–	−1194.312	−54,369.347
Universal two-child (20%)	2022	2028	−8.509	−72.060	1	1	− 719.567	−49,657.684
Universal two-child (40%)	2022	2028	−5.969	−15.930	0	0	−633.924	−48,569.366
Universal two-child (60%)	2022	2029	−3.395	− 219.239	0	1	− 548.235	−47,437.963
Universal two-child (80%)	2022	2029	−0.786	− 149.025	0	0	− 461.277	−46,259.073
Universal two-child (100%)	2023	2029	−12.464	−77.773	1	0	− 372.991	−45,029.947

### Robustness test

To test the stability of the research results, the study carried out a robustness test on the main parameters involved in the model without changing the basic assumptions in this paper, including three items: per capita financing standard, per capita compensation expenditure, and bank interest rate. In the process of robustness testing, the study will increase or decrease by 1% of the per capita financing standard of the URRBMI, per capita compensation expenditure, and bank interest rate at the current level. The results of different situations all showed that, increasing or decreasing the value of the main parameters of the model may change the time point of occurrence and scale of the deficit of the URRBMI fund to a certain extent, and the higher the willingness to give birth under the universal two-child policy, the more obvious the impact, but these does not affect the basic conclusion of this study. The calculation results are shown in Table 5.

**Table 5:** Summary of robustness analysis of main parameter changes Unit: year

Changes			Time point of occurrence of deficit under the strict one-child policy	Universal two-child- 20%	Universal two-child- 40%	Universal two-child- 60%	Universal two-child- 80%	Universal two-child- 100%
Per capita financing standard	+ 1%	Current deficit	2032	3	4	5	6	6
		Accumulated deficit	2040	4	5	6	7	8
	−1%	Current deficit	2021	0	0	0	0	0
		Accumulated deficit	2021	0	0	0	0	0
Per capita compensation expenditure	+ 1%	Current deficit	2021	0	0	0	0	0
		Accumulated deficit	2024	0	0	0	0	0
	−1%	Current deficit	2028	5	7	10	–	–
		Accumulated deficit	2035	8	11	–	–	–
Bank interest rate	+ 1%	Current deficit	2021	1	1	1	1	2
		Accumulated deficit	2027	1	1	2	2	2
	−1%	Current deficit	2021	1	1	1	1	2
		Accumulated deficit	2027	1	1	2	2	2

**Note:** The numbers represent the change value, which appeared, when the occurrence time of the current deficit and accumulated deficit under certain circumstances compared with the occurrence time of deficit under the strict one-child policy. Moving backward is positive and moving forward is negative. “-” means that no funds deficit will occur during the forecast period

## Discussion

This study used the dynamic actuarial models of the URRBMI fund to research the effect of the adjusted universal two-child policy on improving the sustainability of the URRBMI fund. We also examined changes to the ageing population dynamics after adjusting the fertility policy. The results showed that all the key assumptions passed the robustness test. As mentioned in the literature review, few scholars in China have conducted in-depth research on the relationship between fertility policy adjustments and UEBMI fund sustainability [22–24]. On this basis, this study took China’s URRBMI as the research object and examined the link between the adjustment of fertility policies and the sustainability of URRBMI funds.

The results of this study once again confirmed and augmented the arguments of the aforementioned scholars [22–24]: for either UEBMI or URRBMI, fertility policy adjustments can promote the sustainability of medical insurance funds to a certain extent. The results of this study showed that the universal two-child-40% policy, compared to the original policy, can delay the accumulated deficit emergence of URRBMI funds to a certain extent for one year. Moreover, this policy would also have an effect on the ageing of the population of URRBMI participants. Compared with the effect under the original policy, the proportion of the population aged 65 and over in the URRBMI would drop to 19.01% in 2050, a decrease of nearly 5.91%, and the number of URRBMI participants would far exceed the number of participants under the original policy.

The findings of this study are somewhat surprising in that an increase in fertility intentions of couples of childbearing age in accordance with the policy is a prerequisite for more effective outcomes of the universal two-child policy. A possible explanation for this might be that an increase in the willingness to give birth would further increase the total fertility rate, thereby increasing the number of URRBMI participants, improving the age structure among participants, increasing income, and improving the financial operation of the fund. As the results show, if the intention to give birth under the universal two-child policy can reach 80%, it is predicted that the annual number of URRBMI participants will exceed 1.2 billion by 2029. The proportion of the population 65 years old and above will also rise slowly and begin to show a downward trend in 2047. Moreover, in terms of the improvement of fund operation capabilities, compared with the universal two-child-40% policy, the occurrence of accumulated deficit will be further postponed from 2028 to 2029. The adjustment of the universal two-child policy can indeed enhance the sustainable operation of China’s URRBMI fund and delay the occurrence of a fund deficit. Moreover, as couples’ intention to give birth increases, the effect improves. However, due to the slow improvement of intention to give birth, the Chinese government further proposed to implement the policy of allowing each couple to have three children and supporting measures.

In addition, this study compared the changes in the population age structure and the operation of the medical insurance fund between forecast periods of 2021–2030 and 2021–2050 and found that fertility policy adjustments have a certain lag effect, and the effect of the universal two-child policy will take a long time to become fully evident.

## Conclusion

The adjustment of China’s universal two-child policy has played a positive role in improving the sustainability of the URRBMI fund and alleviating the ageing of the population. This adjustment can delay the occurrence of a URRBMI fund deficit and reduce the deficit amount. As the intentions of couples of childbearing age to give birth increases, the effect will improve. However, because the adjustment of the fertility policy has a certain lag, its effect takes a long time to appear. And the forecasts of URRBMI fund income and expenditure in this study are conservative estimates based on the parameters of the NCMS. Therefore, the actual risk of the current operation of the URRBMI fund may be greater than expected. We believe that under the current trend of the rapidly ageing population, the medical needs of the elderly are rapidly growing, and the sustainable operation of the medical insurance fund is facing tremendous pressure. Therefore, multiple measures must be taken to improve the sustainable operation of the URRBMI fund, as follows.

First, China’s provinces (autonomous regions and municipalities) should pave the way for the smooth implementation of the universal two-child policy and three-child policy in light of each region’s actual conditions. To increase fertility desire, it is possible to reduce the financial burden and mental stress on couples of childbearing age and increase maternity benefits by providing financial subsidies, reducing personal taxes, or extending vacation time to ensure the effects of implementation of the fertility policy.

Second, in the face of the rapidly ageing population and the limitation of URRBMI fundraising, it is essential to control the growth rate of medical expenses and improve the efficiency of fund expenditures. The reform of medical insurance payment methods is undoubtedly a powerful method to accomplish this. Particularly for medical services, payment method reform should start with doctors, who are medical service providers, and should be value-oriented. Adopting a compound payment method that combines the global budget, diagnostic-related groups and other payment methods can encourage doctors to independently increase the awareness of controlling fees.

Third, considering that the URRBMI fund may have deficit risks in the short term, it is difficult to maintain the URRBMI fund balance by its own funding mechanism, so more financial support is needed. In the eastern, central, and western regions of China, the ratio of personal and financial financing should be reasonably adjusted in light of each region’s actual conditions. We recommend actively exploring a dynamic financing mechanism that is compatible with China’s economic development level and is linked to the income levels of urban and rural residents. Moreover, to address the continuous increase in medical consumption caused by the rapidly aging population, it is possible to enhance the value preservation and appreciation function of the URRBMI fund or comprehensively postpone the statutory retirement.

In addition, due to the vast territorial extent of China, various provinces (cities, districts) may have different levels of economic development and cultural customs [33, 34], and there may be some differences in the level of fertility desire among various regions and in medical insurance fund income and expenditure. It is difficult to guarantee that the calculation results of this study can be fully applicable to the specific conditions of each region in China or whether these findings will be applicable in the coming decades. In this regard, more in-depth research is needed, such as targeted research based on more detailed provincial samples or conducting the regular follow-up and improvement of national-level data.

## Data Availability

If there is a reasonable need, please contact the corresponding author for data requests.

## Abbreviations

UEBMI: urban employees’ basic medical insurance
NCMS: new rural cooperative medical care system
URBMI: urban residents’ basic medical insurance
URRBMI: urban and rural residents’ basic medical insurance
